# Evaluation of protective efficacy of three novel H3N2 canine influenza vaccines

**DOI:** 10.18632/oncotarget.21104

**Published:** 2017-09-20

**Authors:** Liqing Tu, Pei Zhou, Lutao Li, Xiuzhen Li, Renjun Hu, Kun Jia, Lingshuang Sun, Ziguo Yuan, Shoujun Li

**Affiliations:** ^1^ College of Veterinary Medicine, South China Agricultural University, Guangzhou, Guangdong Province 510642, People's Republic of China; ^2^ Guangdong Provincial Key Laboratory of Prevention and Control for Severe Clinical Animal Diseases, Guangzhou, Guangdong Province 510642, People's Republic of China; ^3^ Guangdong Provincial Pet Engineering Technology Research Center, Guangzhou, Guangdong Province 510642, People's Republic of China

**Keywords:** canine influenza vaccine, H3N2, pVAX1-HA, rCAV2-HA, inactivated CIV

## Abstract

Canine influenza virus (CIV) has the potential risk to spread in different areas and dog types. Thus, there is a growing need to develop an effective vaccine to control CIV disease. Here, we developed three vaccine candidates: 1) a recombinant pVAX1 vector expressing H3N2 CIV hemagglutinin (pVAX1-HA); 2) a live attenuated canine adenovirus type 2 expressing H3N2 CIV hemagglutinin (rCAV2-HA); and 3) an inactivated H3N2 CIV (A/canine/Guangdong/01/2006 (H3N2)). Mice received an initial intramuscular immunization that followed two booster injections at 2 and 4 weeks post-vaccination (wpv). The splenic lymphocytes were collected to assess the immune responses at 6 wpv. The protective efficacy was evaluated by challenging H3N2 CIV after vaccination (at 6 wpv). Our results demonstrated that all three vaccine candidates elicited cytokine and antibody responses in mice. The rCAV2-HA vaccine and the inactivated vaccine generated efficient protective efficacy in mice, whereas limited protection was provided by the pVAX1-HA DNA vaccine. Therefore, both the rCAV2-HA live recombinant virus and the inactivated CIV could be used as potential novel vaccines against H3N2CIV. This study provides guidance for choosing the most appropriate vaccine for the prevention and control of CIV disease.

## INTRODUCTION

In 2004, canine influenza virus (CIV) was first reported in Florida (United States of America, USA) [1]. CIV originated from the equine influenza virus (EIV) H3N8. However, H3N2 CIV of avian origin was subsequently identified in 2007 among dogs in South Korea [2] and was further traced back to 2006 when it was identified in China according to our previous study [3]. In 2015, a H3N2 CIV outbreak occurred in Chicago, and the virus then rapidly spread to numerous states in the USA [4, 5]. And, seroepidemiological evidence of H3N8 CIV among pet dogs was reported in China according to our previous study [6].

CIV, mainly including H3N8 [1, 7, 8] and H3N2 [2, 9], naturally or artificially infects numerous species, such as horses [10], cats [11], mice [12], ferrets [13], and pigs [14]. Given that CIV represents a potential risk for interspecies and crosspieces transmission [15], there is a growing concern regarding the epidemic threat that CIV poses. Indeed, the number of cases of natural H3N2 CIV infection in dogs has been increasing yearly [9, 16, 17], increasing the risk of viral exposure to animal populations or humans via infected animals. Therefore, the prevention and control of CIV disease is necessary for animal health and public health concerns.

Over recent decades, influenza A virus vaccines have been developed to contain several viral strains, and different vector types have been used for delivery. The most common human influenza A vaccine contains an inactivated virus [18–20]. Furthermore, multiple antigenic peptide-based vaccines [21–23], live attenuated influenza virus [24–27], recombined adenovirus [28–30], and DNA vaccines [31, 32] that express one or more viral antigens [33] have demonstrated protective efficacy against influenza.

In an attempt to develop a vaccine against CIV, a canarypox-vectored EIV H3N8 vaccine expressing hemagglutinins of A/equine/Kentucky/94 (vCP1529) and A2/equine/Ohio /03 (vCP2242) and an equine herpesvirus type 1 recombinant EIV H3N8 vaccine expressing hemagglutinins of A/canine/PA/10915-07 were evaluated against H3N8 CIV in dogs [34, 35]. In addition, the inactivated H3N2 CIV vaccine (A/canine/Korea/02/07) provides protection against H3N2 CIV infection in dogs [36]. However, only commercial inactivated H3N8 CIV and H3N2 CIV vaccines are approved in the USA [37]. In this study, we generated and evaluated three H3N2 CIV vaccine candidates, including a recombinant DNA vaccine, recombinant canine adenovirus, and an inactivated CIV vaccine. Here, we assessed the immunogenicity and protective efficacy elicited by each vaccine in mice. This study provides guidance for choosing the most appropriate vaccine platform for the prevention and control of H3N2 CIV disease.

## RESULTS

### CIV vaccine candidates were successfully prepared

**Figure 1:**
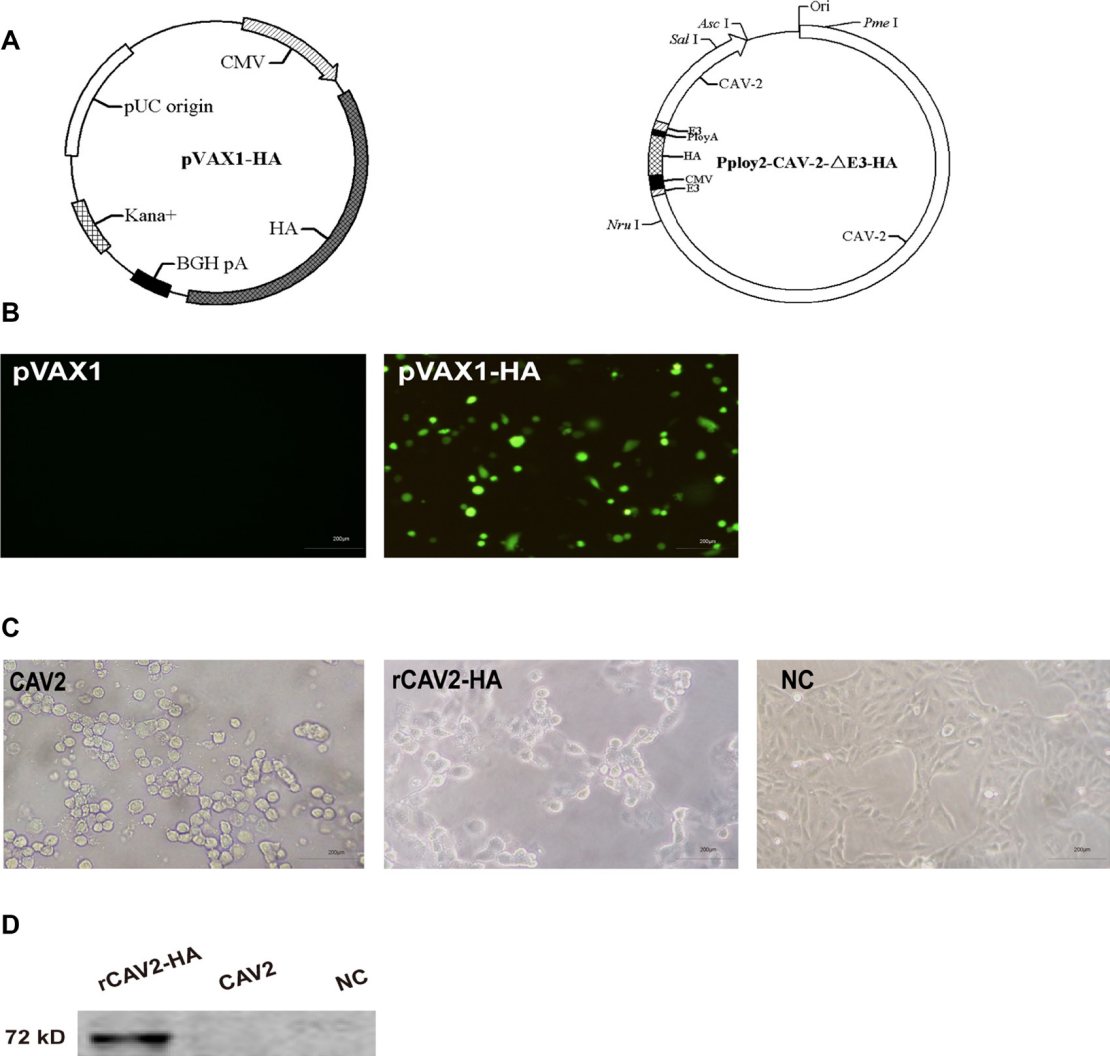
Construction and identification of the pVAX1-HA plasmid and recombined CAV2-HA The hemagglutinin (HA) gene fragment of the canine influenza virus (A/canine/Guangdong/01/2006 (H3N2)) was inserted into the pVAX1 and ppoly2-CAV2-ΔE3 vectors (**A**). HA expression from the pVAX1-HA vector and rCAV2-HA was detected by indirect immunofluorescence and Western blot, respectively (**B**, **D**). In addition, MDCK cells exhibited grape-like gatherings indicative of an adenovirus infection after infection with rCAV2-HA for 24 h (**C**).

Figure 1A demonstrates that the HA gene from CIV (A/canine/Guangdong/01/2006 (H3N2)) was favorably inserted into the pVAX1 and ppoly2-CAV2-ΔE (the partial E3 region of canine adenovirus type 2) vectors. The recombined pVAX1-HA vector was transferred into Madin-Darby canine kidney (MDCK) cells for 72 h. HA expression from the pVAX1-HA vector was detected by an indirect immunofluorescence test (Figure 1B) and PCR analysis of MDCK cells (data not shown). In addition, MDCK cells elicited the typical cytopathic effect of an adenovirus infection after infection with rCAV2-HA (recombined CAV2-HA virus) for 24 h (Figure 1C), as characterized by the grape-like gathering of cells adjacent to the infected cells. HA expression by rCAV2-HA was verified by Western blot (Figure 1D).

### HI assay

At two, four, six, and eight weeks after the initial vaccination, serum samples were collected for antibody assessment. Samples from mice vaccinated with pVAX1-HA, rCAV2-HA, and an inactivated CIV vaccine all exhibited an antibody response against CIV as defined by a hemagglutination inhibition (HI) titer ≥ 2^4^ (Table 1). As expected, the HI titers of the control groups vaccinated with CAV2, pVAX1 vector, adjuvant alone, or phosphate buffered solution (PBS) were negative (< 1:2) (Table 1).

**Table 1:** HI antibody titers were detected at 2, 4, 6 and 8 wpv

		HI			
		2 wpv	4 wpv.	6 wpv	8 wpv
Group 1	pVAX1-HA	8	25.40	32^a,b^	12.70
Group 2	rCAV2-HA	32	41.78	98.05^a,c^	25.40
Group 3	Inactivated vaccine	32	47.95	120.82^b,c^	40.32
Group 4	pVAX1	< 2	< 2	< 2	< 2
Group 5	CAV2	< 2	< 2	< 2	< 2
Group 6	Adjuvant	< 2	< 2	< 2	< 2
Group 7	PBS	< 2	< 2	< 2	< 2
Group 8	NI	< 2	< 2	< 2	< 2

Notes:

Data are presented as geometric mean titer (*n* = 12, at 2, 4 and 6 wpv; *n* = 3, at 8 wpv).

Significant or extremely significant difference at *p* < 0.05 or *p* < 0.01, respectively.

NI = No immunization

CAV2-HA = live recombinant canine adenovirus 2 (CAV2) vector expressing the H3 hemagglutinin protein from the virus strain A/canine/Guangdong/01/2006(H3N2).

CAV2 = empty CAV2 vector

wpv = weeks post-vaccination

^a,b,c^ The same letter indicates the intergroup difference analysis.

^a^The difference is extremely significant (*p* < 0.01); ^b^The difference is extremely significant (*p* < 0.01); ^c^The difference is not significant (*p* > 0.05).

### Lymphocyte proliferation and cytokine levels

**Figure 2:**
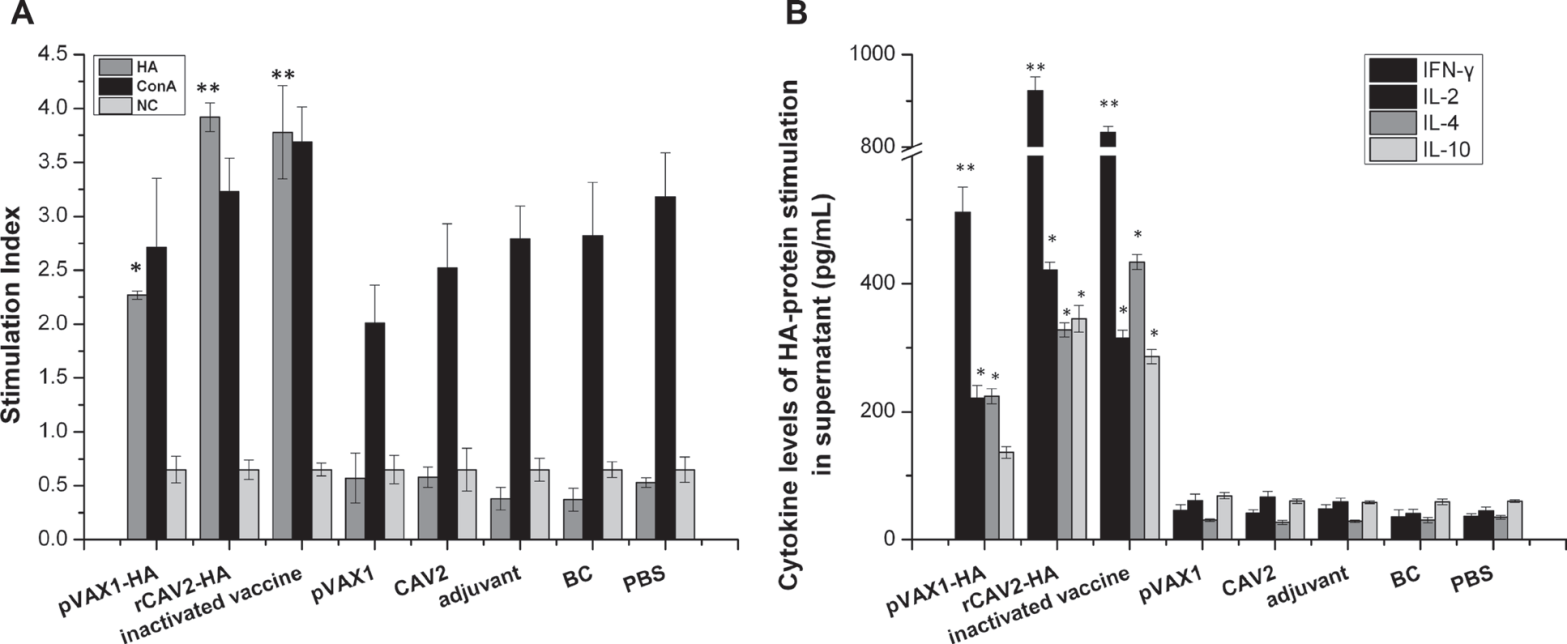
Splenic lymphocyte proliferation and cytokine secretion assays At 6 wpv, the splenic lymphocytes from all mice (*n* = 3) were stimulated with antigen (HA protein and concanavalin A) after 72 h. The SI was calculated as the ratio of the average OD_450_ value of wells containing antigen-stimulated cells to the mean OD_450_ value of wells containing only cells with medium (**A**). The secretion of cytokines against HA protein was measured via ELISA (**B**). Data are presented as the mean ± SD. The difference is significant (^*^p < 0.05) and extremely significant (^**^p < 0.01) compared with the related control group (inoculated mice with empty plasmid, adjuvant and PBS).

Splenic lymphocyte proliferation in mice (*n* = 3/group) was detected using a CCK-8 kit at 6 wpv. The stimulation index (SI) revealed prominent increases in lymphocytes in mice vaccinated with pVAX1-HA, rCAV2-HA, and inactivated vaccine (Figure 2A). Compared with the negative control, the SI of the splenic lymphocytes of mice vaccinated with the pVAX1-HA DNA vaccine, the live rCAV2-HA and the inactivated vaccine were significantly increased to 3.49-, 6.03- and 5.81-fold, respectively.

In addition, splenic lymphocyte cultures were collected to quantify the level of cytokine production after HA protein antigen stimulation for 72 h. All cytokine levels (interleukin (IL)-2, IL-4, IL-10 and interferon (IFN)-γ) in mice vaccinated with pVAX1-HA, rCAV2-HA, and inactivated CIV were significantly increased compared with mice vaccinated with empty plasmid, adjuvant or PBS (*p* < 0.05) (Figure 2B).

### All CIV vaccines exhibit protective efficacy against CIV infection

**Figure 3:**
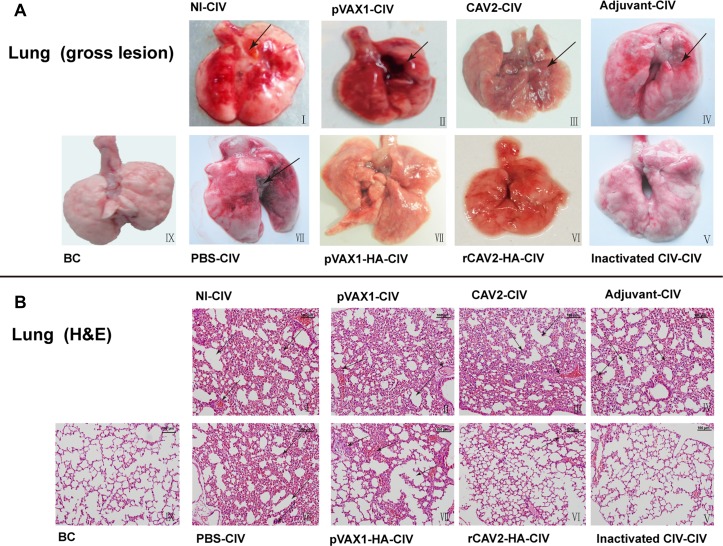
Histopathology (200×) Lung lesions in mice at 5 dpc. No immunization and CIV challenge (I), pVAX1 vector vaccination and CIV challenge (II), CAV2 vaccination and CIV challenge (III), adjuvant inoculation and CIV challenge (IV), inactivated CIV vaccination and CIV challenge (V), rCAV2-HA vaccination and CIV challenge (VI), pVAX1-HA DNA vaccination and CIV challenge (VII), PBS inoculation and CIV challenge (VIII), and blank control (IX).

The rectal temperature of mice was measured every day post-challenge. However, a relatively constant temperature of 36.8 ± 0.2°C was maintained (data not shown). At 5 days post-challenge (dpc), lungs were collected for pathology. Gross lung lesions (hemorrhages and tumidness) were characterized in all control groups (Figure 3A). The lungs also exhibited severe and extensive histopathologic changes (hematoxylin and eosin (HE) stain) (Figure 3B). Specifically, the alveolar septa were thickened, and the alveolar lumen was infiltrated with neutrophils and other inflammatory cells (Figure 3A. I~IV. In addition, lung sections from challenged mice vaccinated with pVAX1-HA exhibited mild pathology with only a moderate thickening of the alveolar septa in some instances (Figure 3B.VII). Lung lesions in mice vaccinated with rCAV2-HA exhibited moderate pathology (Figure 3B.VI) that included a partial bronchus filled with small neutrophils. Remarkably, the lungs of the group vaccinated with inactivated vaccine (Figure 3AV) exhibited no difference compared with the unchallenged control group (Figure 3A.IX).

**Figure 4:**
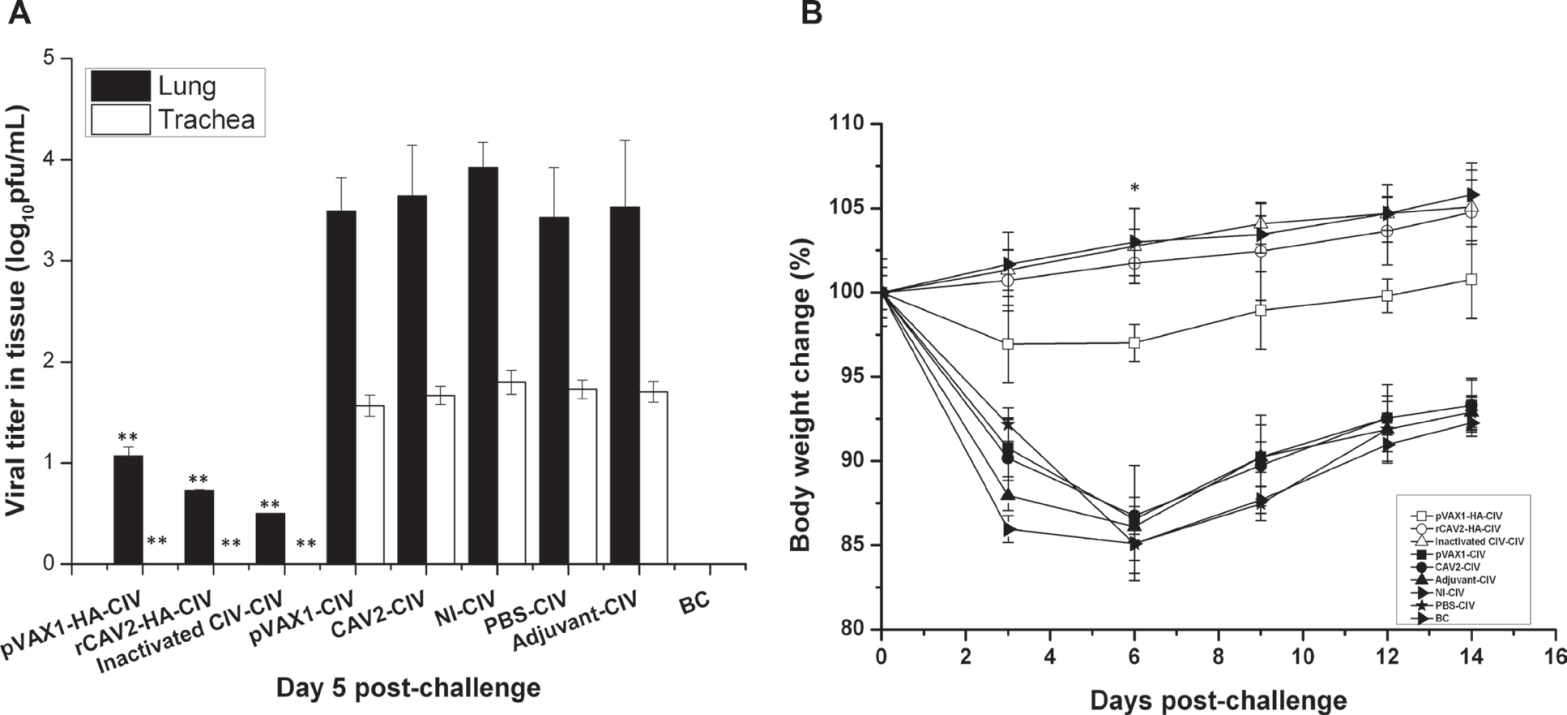
Virus load and change in body weight Viral titers in the respiratory tract (lungs and tracheas) were detected using a plaque assay on 5 dpc (**A**). On 0, 3, 6, 9, and 14 dpc, the body weight of mice was recorded, and the percent body weight change was calculated (**B**). Data are presented as the mean ± SD and analyzed as noted above.

In Figure 4A, the virus titers of lungs and tracheas from vaccinate mice were significantly reduced compared with control mice, suggesting that all candidate vaccines were protective against virus replication in the respiratory tract (Figure 4A).

The percent of weight change was based on the starting weight on 0 dpc. In this trial body weight loss was not noted in mice from the rCAV2 and inactivated groups, but slight body weight loss was noted in mice from the pVAX1-HA DNA group. However, significant body weight loss was noted in mice in the no vaccination control groups (pVAX1 group, CAV2 group, adjuvant group, NI group and PBS group), and the body weight loss rate exhibited its lowest point on 6 dpc (Figure 4B).

## DISCUSSION

A hallmark of the influenza virus is the remarkable variability of its major surface glycoproteins. HA is one such surface glycoprotein that has been used in recombinant vaccines against influenza because it is a dominant antigen that can enhance the immune response to influenza [38]. The HA amino acid sequence of the A/canine/Guangdong/01/2006 (H3N2) virus used in this study was 97.2% ~98.9% similar to 16 other H3N2 CIV strains isolated in China and Korea, indicating that this strain has the potential to be a universal vaccine to against H3N2 CIVs. Mice have been used as a traditional animal model for influenza virus infection and vaccination [39]. In this study, the immunogenicity and protective efficacy were analyzed by vaccinating mice with three types of CIV vaccines, including a pVAX1-HA DNA vaccine, a live rCAV2-HA vaccine, and an inactivated vaccine.

As previously reported, the plasmid pVAX1 serves as a promising approach for antigen preparation in vaccine development. This DNA vaccine vector system is ideal for high-level recombinant protein production in CHO cells [40]. Additionally, as a recombinant viral vector, CAV2 provides a safe and long-term protective effect in veterinary fields [41] that reduced the natural infection risk [42]. An inactivated viral-based vaccine is a traditional vaccine platform that is used to against a wide range of infections. Montanide^TM^ Gel01 ST was used as adjuvants for both inactivated and live virus vaccines [43, 44] to enhance the protective efficacy in animals.

Of note, we observed that the HI titers of the rCAV2-HA group were increased compared with the pVAX1-HA group but reduced compared with the inactivated group at the same time post vaccination (Table 1). This result reveals that the more CIV-specific antibodies for humoral immunity were produced by inactivated virus vaccination compared with other vaccines. The pVAX1-HA DNA group, which was exposed to a DNA vaccine, maintained an acceptable level of humoral immunity; however, the HI titers were reduced compare with the other two vaccine groups. The antibodies are effective against CIV infection and contribute to viral clearance in the organs.

IL-2 and IFN-γ are produced by Th1 cells to promote viral clearance by stimulating proliferation and cytotoxic T lymphocyte responses [45]. IL-4 is involved in a Th2 response, and IL-10 produces an anti-inflammatory response. Antigen-specific cellular responses were characterized based on the balance of Th2 and Th1 cytokines [46]. Cytokine secretion in the pVAX1-HA, rCAV2-HA and inactivated groups were increased, and the rCAV2-HA group exhibited the highest levels. This result suggests that the T cell-mediated immune response was activated, and live recombinant virus (rCAV2-HA group) stimulates an increased response compared with the inactivated virus vaccine (inactivated group) and the DNA vaccine (pVAX1-HA group). One explanation for this finding involves complete antigen epitope stimulation from live virus. In the splenic lymphocyte proliferation assay, the group with the highest SI increase was also the rCAV2-HA group given the persistent antigen presentation of live virus. This result implies that the lymphocytes specific to CIV HA protein were significantly responsive.

Histopathological lesions were detected in the respiratory tracts (trachea and lung) of CIV-challenged mice, which was consistent with the previous study [12]. We found that both the rCAV2-HA and the inactivated vaccine provided considerable protection against lung pathology and completely prevented weight loss following CIV challenge. However, some lung pathology and body weight loss were noted in mice receiving pVAX1-HA vaccination. However, the virus load was minimally detected after exposure to the three candidate vaccines. Previous research confirmed that H3N8 CIV (equine origin) resulted in an average loss of 5% body weight at 5 dpc [47].

Our results demonstrated that a live recombinant virus (rCAV2-HA) and an inactivated vaccine generated significant levels of immune activation in mice. Additionally, all three candidate vaccines displayed heightened protective efficacy against CIV H3N2 challenge, whereas limited protection was provided by the pVAX1-HA DNA vaccine. Therefore, both the rCAV2-HA live recombinant virus and the inactivated CIV could be used as potential novel vaccines against the current epidemic of CIV H3N2. Moreover, the time of challenge with H3N2 CIV after vaccination was 2 wpv. If the time of challenge was extended, a stronger immune response may be activated that would provide better protection from virus. In addition, a potential limitation should be noted. These CIV vaccines were only evaluated in mice, and the further studies in dogs should be explored.

## MATERIALS AND METHODS

### Cells, virus and vectors

MDCK cells (ATCC CRL-2936; American Type Culture Collection [ATCC], Manassas, VA) were maintained in Eagle's Minimum Essential Medium with 10% fetal bovine serum (FBS) and an antibiotic solution (10,000 IU/mL penicillin and 10,000 μg/ mL streptomycin). All cells were incubated at 37°C and 5% CO_2_. The avian-origin CIV strain A/canine/Guangdong/01/2006 (H3N2) was used for the inactivated vaccine and challenge experiment [3]. The pVAX1 vector (Invitrogen, USA), a eukaryotic expression plasmid, was approved for recombined DNA vaccine and used according to the manufacturer's protocol [48]. The ppoly2-CAV2-ΔE3 vector is mainly composed of the partial E3 region of CAV-2, human cytomegalovirus (hCMV) immediate-early gene promoter, polyA and the SV40 early mRNA polyadenylation signal. This vector was used for the recombined vaccine [49].

### Mice

A total of 96 specific pathogen-free female BALB/c mice (six weeks old) were produced by the Center for Laboratory Animal Science of Guangdong and used for this study. All animal studies were conducted under guidelines approved by the Animal Care and Use Committee of the South China Agricultural University.

### Vaccines preparation

The HA gene fragment amplified from the CIV genome was inserted into the pVAX1 vector to construct the recombinant plasmid pVAX1-HA, which was transferred into MDCK cells using Lipofectamine^®^ 2000 Transfection reagent (Invitrogen). The expression of HA mRNA and protein in MDCK cells were detected by RT-PCR and indirect immunofluorescence assays (IFA). The pVAX1-HA plasmid used for vaccination was extracted from *E. coli* DH5α using a no-endotoxin plasmid extract kit (TIANGEN, CHINA).

In addition, we generated the recombinant canine adenovirus type 2 virus (rCAV2-HA) expressing H3N2 CIV HA protein as previously described [49]. Briefly, the HA gene (A/canine/Guangdong/01/2006 (H3N2)) was inserted into the ppoly2-CAV2-ΔE3 vector (ppoly2-CAV2-ΔE3-HA). The chains and rings (1:1 ratio) were co-transferred into MDCK cells using Lipofectamine^®^ 2000 Transfection reagent, and the cells were cultivated at 37°C for 72 h. Subsequently, the recombined ppoly2-CAV2-ΔE3-HA virus (rCAV2-HA) was collected and identified based on adenovirus cytopathologic characteristics as assessed by electron microscopy. HA protein expression in MDCK cells was verified by Western blotting, and the rabbit anti-H3N2 HA monoclonal antibody (obtained from our Lab) and IRDye 680RD goat (polyclonal) anti-rabbit IgG (LICOR, USA) were used. The viral titer of rCAV2-HA and CIV were quantified by TCID_50_ in MDCK cells as previously described [50, 51]. The virus used for the vaccination experiment was used to inoculate MDCK cells for viral amplification. The virus was purified by sucrose density gradient centrifugation before inactivation. The virus was inactivated with 0.05% formaldehyde [52] and assessed by performing serial passages in eggs according to the protocol published in European Pharmacopeia [53]. Inactivated CIV was inoculated in mice with an adjuvant, such as Montanide GEl 01st (SEPPIC, France), at a ratio of 80:20 by volume (according to the manufacturer's recommendations).

### Immunization

The procedure for the immunization experiment is presented in Supplementary Figure 1. Mice were randomly divided into eight groups (*n* = 12 per group) and were intramuscularly injected with vaccines as follows: Group 1 (pVAX1-HA group), 100 μg protein pVAX1-HA in 0.1 mL; Group 2 (rCAV2-HA group), rCAV2-HA (10^4^ TCID_50_) with 20 μL adjuvant in 0.1 mL; Group 3 (inactivated group), inactivated CIV (10^4^ TCID_50_) with adjuvant in 0.1 mL; Group 4 (pVAX1 group):, 100 μg protein pVAX1 in 0.1 mL; Group 5 (CAV2 group): CAV2 (10^4^ TCID_50_) with 20 μL adjuvant in 0.1 mL; Group 6 (adjuvant group), 0.1 mL adjuvant; Group 7 (PBS group), 0.1 mL PBS; Group 8 (NI group), no immunization; Blank control group (BC), no immunization and no challenge.

Mice received an initial intramuscular immunization that was defined as 0 wpv. Then, mice received a booster immunization at 2 and 4 wpv. Mice were intramuscularly injected with an equal volume into each calf muscles.

### HI assay

At two, four, six, and eight weeks after the initial vaccination, serum samples were collected from the tail vein of all mice to evaluate the HI antibody and cytokine expression levels. Sera were heat-inactivated (at 56°C for 30 min) and treated with a receptor-destroying enzyme (RDE) (1:3) before the test. The HI assay was performed as described previously [54].

### Lymphocyte proliferation and cytokine production assay

Briefly, splenocyte suspensions were isolated from mice (3/group) by pushing the spleens through a wire mesh one day prior to the challenge. RBCs were removed using the RBC lysis solution (Sigma), and splenocytes were supplemented with RPMI-1640 medium including 20% FBS and an antibiotic solution (10,000 IU/mL penicillin and 10,000 μg/mL streptomycin). Cells were plated in 96-well flat-bottom plates at 100 μL/well (5 × 10^5^ cells/well). Subsequently, 100 μL/well of medium containing HA protein (10 μg/mL), PBS (negative control) or Concanavalin A (ConA, 5 μg/mL; Sigma) (positive control) was added at 37°C and 5% CO_2_ for 72 h. The proliferative activity was measured using a CCK8 kit (Beyotime, CHINA). The SI was calculated as the ratio of the average OD_450_ value of the wells containing antigen-stimulated cells to the average OD_450_ value of the wells containing only cells with the medium. All assays were performed in triplicate. Passive secretion of cytokines (interleukin (IL)-2, IL-4, IL-10, and interferon (IFN)-γ) from collected supernatants was detected using specific ELISA kits (R&D). Procedures were performed per the manufacturer's instructions. For each plate, the OD_450_ was transformed to a concentration by applying a linear regression formula, which was calculated from the standards of each kit.

### Challenge

Mice (*n* = 6/group, other 3 mice as blank control for each group) were anesthetized with isoflurane (3%) and were intranasally inoculated with 0.1 mL CIV (10^5^ TCID_50_) at 6 wpv. The body weight was monitored and recorded on 0, 3, 6, 9, and 14 dpc. The rectal temperature was measured every dpc. On 5 dpc, 3 mice were euthanized by carbon dioxide inhalation in a gas chamber. After carbon dioxide treatment, heart rates, respiratory rates, and pupillary light reflexes were tested to confirm death. Lungs were collected rapidly and then immersed in 10% neutral formalin buffer overnight to prevent autolysis. The lungs were then sectioned and stained with HE for histopathological analysis. In addition, the virus titers in respiratory tract tissues were assessed by the plaque assay [55]. The remaining 3 mice were euthanized at 8 wpv.

### Statistical analysis

All data were processed and analyzed using SPSS13.0 Data Editor (SPSS Inc., Chicago, IL, U.S.A.). Lymphoproliferation, cytokine production and viral titers were compared between groups using one-way ANOVA. Statistical analysis was performed using the unpaired Student's *t*-test. *p* values of *p* < 0.05 (^*^) and *p* < 0.01 (^**^) were considered statistically significant and highly significant, respectively.

### Ethical

All animal care and experimental procedures were performed under an approved protocol in compliance with CDC Institutional Animal Care and Use Guidelines.

## SUPPLEMENTARY MATERIALS FIGURE



## Abbreviations

(CIV): Canine influenza virus
(USA): United States of America
(EIV): Equine influenza virus
(PBS): Phosphate buffered solution
(MDCK): Madin-Darby canine kidney
(FBS): Fetal bovine serum
(hCMV): Human cytomegalovirus
(HI): Hemagglutination inhibition assay
(wpv): Weeks post-vaccination
(dpc): Days post-challenge
(RDE): Receptor-destroying enzyme
(RBC): Red blood cells
(IFN): Interferon
(IL): Interleukin
(HE): Hematoxylin and eosin
(SI): Stimulation index
