# Supplementary figures and images for: Evaluation of protective efficacy of three novel H3N2 canine influenza vaccines
Source: Oncotarget. 2017 Sep 20;8(58):98084–93. doi: 10.18632/oncotarget.21104 (PMC5716715; doi:10.18632/oncotarget.21104)

## SUPPLEMENTARY MATERIALS

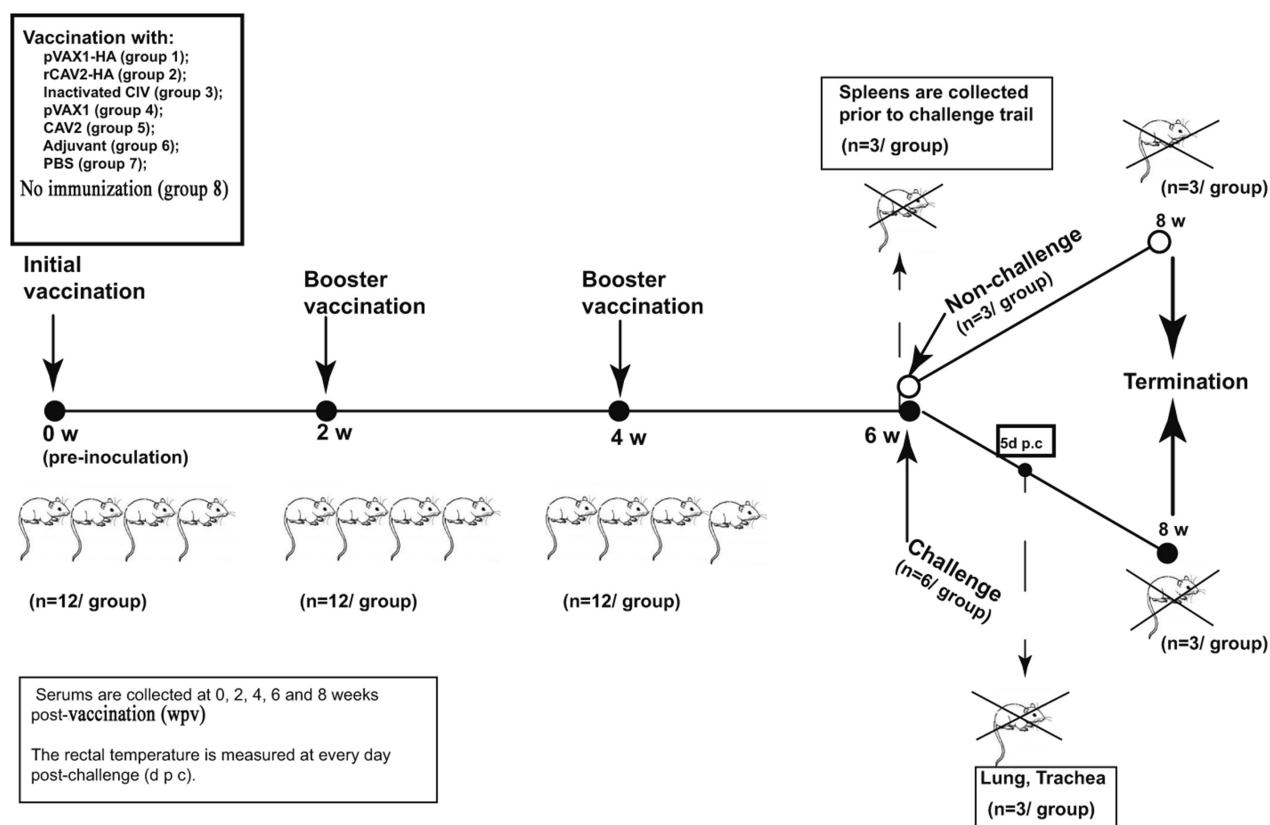

**Supplementary Figure 1: Diagram of the experimental design.**

Supplement: Supplementary file 1 [file oncotarget-08-98084-s001.pdf]
